# In-depth analysis of the chicken egg white proteome using an LTQ Orbitrap Velos

**DOI:** 10.1186/1477-5956-9-7

**Published:** 2011-02-07

**Authors:** Karlheinz Mann, Matthias Mann

**Affiliations:** 1 Max-Planck-Institut für Biochemie, Abteilung Proteomics und Signaltransduktion, Martinsried, Germany

## Abstract

**Background:**

Hen's egg white has been the subject of intensive chemical, biochemical and food technological research for many decades, because of its importance in human nutrition, its importance as a source of easily accessible model proteins, and its potential use in biotechnological processes. Recently the arsenal of tools used to study the protein components of egg white has been complemented by mass spectrometry-based proteomic technologies. Application of these fast and sensitive methods has already enabled the identification of a large number of new egg white proteins. Recent technological advances may be expected to further expand the egg white protein inventory.

**Results:**

Using a dual pressure linear ion trap Orbitrap instrument, the LTQ Orbitrap Velos, in conjunction with data analysis in the MaxQuant software package, we identified 158 proteins in chicken egg white with two or more sequence unique peptides. This group of proteins identified with very high confidence included 79 proteins identified in egg white for the first time. In addition, 44 proteins were identified tentatively.

**Conclusions:**

Our results, apart from identifying many new egg white components, indicate that current mass spectrometry technology is sufficiently advanced to permit direct identification of minor components of proteomes dominated by a few major proteins without resorting to indirect techniques, such as chromatographic depletion or peptide library binding, which change the composition of the proteome.

## Background

The avian egg white functions as a shock-absorber, keeps the yolk in place, constitutes an antimicrobial barrier, and provides water, protein and other nutrients to the developing embryo. Besides these biological roles it is an inexpensive source of high quality protein for food industries, contains proteins of pharmaceutical interest, and proteins that have found widespread use in biomedical research and protein chemistry [1-6]. Therefore, it is no surprise that egg white has been the target of proteomic studies previously. Raikos et al. [7] used 2D electrophoresis to separate the proteins and MALDI-TOF-based peptide mass fingerprinting to analyze the spots. Seven proteins were identified. 2D electrophoresis, peptide mass fingerprinting and LC-MS/MS using a quadrupole-TOF mass spectrometer were used to identify sixteen proteins in a more advanced study [8]. We have reported the high confidence identification of 78 proteins in egg white using a workflow consisting of SDS-PAGE to separate proteins, coupled to LC-MS/MS and MS^3 ^with an LTQ-FT mass spectrometer [9]. The use of combinatorial hexapeptide libraries [10] in conjunction with LC-ESI-IT-MS/MS allowed the identification of 148 egg white proteins, demonstrating the power of this novel technology to detect minor components even in samples dominated by a few major proteins [11]. Bead-coupled peptide libraries are thought to "equalize" the proteome by providing similar numbers of binding sites to each of the different proteins contained in a proteome. However, it was shown recently that, in contrast to the previously proposed mode of action, the beneficial effect of the peptide beads does not appear to be mediated by specific interaction but is instead dominated by simple hydrophobic effects [12].

Samples, such as egg white, where ovalbumin, ovotransferrin and ovomucoid make up approximately 75% of the total protein, are traditionally difficult to analyze in depth by mass spectrometry, because the peptides of these few proteins tend to dominate the full mass spectra and are selected for fragmentation by MS/MS over and over again. This difficulty has been addressed by the above-mentioned peptide ligand library bead or hydrophobic bead technology [10-12]. However, disadvantages of the peptide library technology include that it is only amenable to soluble proteins and that the composition of the proteome is modified in an unknown and unpredictable way, which makes it impossible to determine the absolute quantity of the proteins. Since the publication of those studies, new developments in instrumentation and peptide identification software occurred, which raised the possibility that in-depth investigation of the egg white proteome would not have to rely on enrichment technologies any more. In the present report we used a novel dual pressure linear ion trap instrument, the LTQ Orbitrap Velos [13]. This new generation of mass spectrometers has increased sensitivity and scan speed as compared to the LTQ-FT used in our previous study [9]. The LTQ Orbitrap Velos is fast enough to isolate and fragment ten or more peaks simultaneously with the acquisition of one high resolution mass full scan spectrum. For evaluation of spectra and database searches we used the MaxQuant software, which is particularly suited for the use of high-resolution MS data and yields very high mass accuracy and peptide identification rates [14-16].

## Materials and methods

### Preparation of peptides

Proteins were separated by PAGE with pre-cast 4-12% Novex Bis-Tris gels in MES buffer, using reagents and protocols supplied by the manufacturer (Invitrogen, Carlsbad, CA). The kit sample buffer was modified by adding SDS and β-mercaptoethanol to a final concentration of 5% and 2%, respectively, and the sample was suspended in 40 μl sample buffer/100 μg of egg white protein and boiled for 5 min. Gels were stained with colloidal Coomassie (Invitrogen) after electrophoresis. Three lanes loaded with 100 μg of protein were used in each of three separate experiments. The gels were cut into 24 slices for in-gel digestion with trypsin [17] and the peptides were cleaned with Stage Tips [18] before mass spectrometric analysis.

### LC-MS and data analysis

Peptide mixtures were analyzed by on-line nanoflow liquid chromatography using the EASY-nLC system (Proxeon Biosystems, Odense, Denmark, now part of Thermo Fisher Scientific) with 15cm capillary columns of an internal diameter of 75 μm filled with 3 μm Reprosil-Pur C18-AQ resin (Dr. Maisch GmbH, Ammerbuch-Entringen, Germany). The gradient consisted of 5-30% acetonitrile in 0.5% acetic acid at a flow rate of 250nl/min for 85min, 30-60% acetonitrile in 0.5% acetic acid at a flow rate of 250nl/min and 60-80% acetonitrile in 0.5% acetic acid at a flow rate of 250nl/min for 7min. The eluate was electrosprayed into an LTQ Orbitrap Velos (Thermo Fisher Scientific, Bremen, Germany) through a Proxeon nanoelectrospray ion source. The LTQ Orbitrap Velos was operated in a CID top 10 mode essentially as described [13]. The resolution was 30,000 (1 experimental data set) and 60,000 (2 experimental data sets) for the Orbitrap whereas fragment spectra were read out at low resolution in the LTQ. Ion trap and orbitrap maximal injection times were set to 25ms and 500ms, respectively. The ion target values were 5000 for the ion trap and 1000000 for the orbitrap. Raw files were processed using version 1.1.0.45 of MaxQuant (http://www.maxquant.org/). For protein identification the ipi.CHICK protein database v3.65 (http://www.ebi.ac.uk/IPI/IPIchicken.html) was combined with the reversed sequences and sequences of widespread contaminants, such as human keratins. Carbamidomethylation was set as fixed modification. Variable modifications were oxidation (M), N-acetyl (protein) and pyro-Glu/Gln (N-term). Initial peptide mass tolerance was set to 7ppm and fragment mass tolerance was set to 0.5 Da. Two missed cleavages were allowed and the minimal length required for a peptide was seven amino acids. Two unique peptides were required for high-confidence protein identifications. These could also be derived from different experimental data sets. The peptide and protein false discovery rates (FDR) were set to 0.01. The maximal posterior error probability (PEP), which is the probability of each peptide to be a false hit considering identification score and peptide length, was set to 0.01. Proteins identified in two of three experimental data sets were accepted. Tentative identifications with only one unique peptide, or two (or more) unique peptides in only one experimental data set, were manually validated considering the assignment of major peaks, occurrence of uninterrupted y- or b-ion series of at least 3 consecutive amino acids, preferred cleavages N-terminal to proline bonds, the possible presence of a2/b2 ion pairs and mass accuracy. The ProteinProspector MS-Product program (http://prospector.ucsf.edu/) was used to calculate the theoretical masses of fragments of identified peptides for manual validation. The exponentially modified protein abundance index (emPAI) provides an estimate of the absolute abundance of a protein from the ratio of observed to observable peptides [19] and was used to differentiate between major and minor proteins. The emPAI calculation considered the preset modifications, miss-cleavages and different charge states. Usually only unique peptides were counted, but in the case of substantial overlap, i.e. almost identical proteins, these were grouped together and the emPAI was calculated for the protein with highest sequence coverage.

## Results and discussion

**Figure 1:**
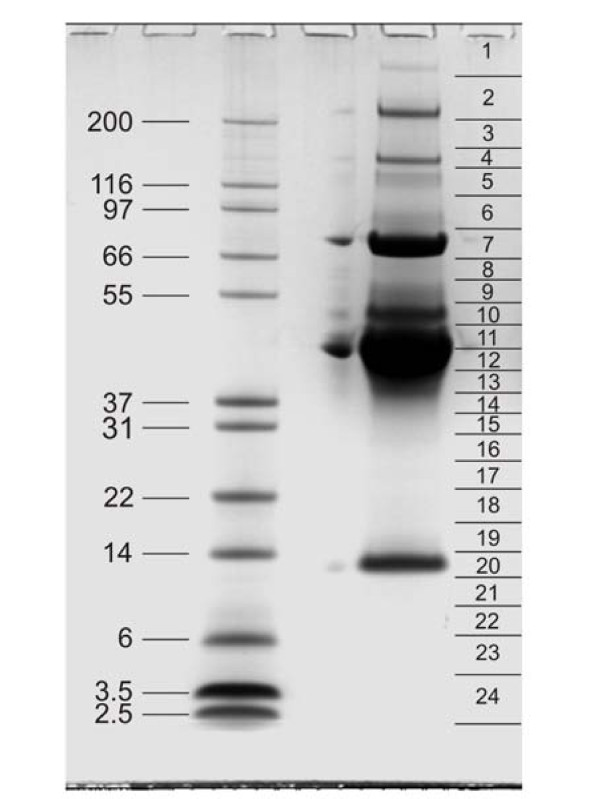
**PAGE separation of chicken egg white proteins**. Left, the marker proteins are labeled with their molecular weight in kDa. Right, slices used for in-gel digestion are indicated. Overloaded gels show additional bands in the low molecular weight region [9].

**Figure 2:**
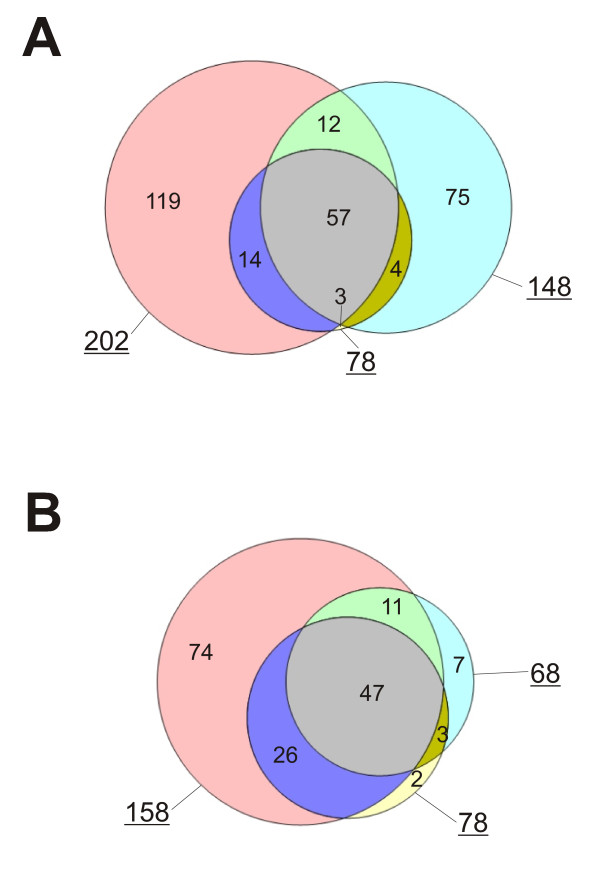
**Overlap between recent egg white proteomic studies**. **A**, total number of identified proteins; **B**, protein identifications with 2 or more sequence unique peptides (or with confirmation by MS^3 ^in [9]). Underlined numbers indicate sum of identified proteins in the present report (202/158), in [11] (148/68) and in [9] (78/78). Venn diagrams were drawn and calculated using the Venn Diagram Plotter of http://omics.pnl.gov/software/VennDiagramPlotter.php.

Egg white proteins were separated by PAGE and gels were cut into 24 sections for in-gel digestion (Figure 1) followed by mass spectrometric analysis of the resulting peptides on a high resolution instrument with fast sequencing speed. Three repetitions of the experiment resulted in seventy-two raw-files that yielded a total of approximately 61,500 peptides identified and accepted with a peptide posterior error probability (PEP) of <0.01 and a preset false discovery rate (FDR) of 0.01. Of these, 1,373 peptides were sequence-unique. The average absolute mass deviation was 1.2ppm. By searching of a chicken protein sequence database and by accepting only protein identifications with two sequence-unique peptides occurring in at least two of three experimental data sets, 158 proteins were identified (Additional file 1: Egg white proteins identified with two or more unique peptides). If approximately equal conditions are used between the present study and the peptide library-based study [11] by also considering proteins identified with single peptides occurring in at least two experimental data sets, or proteins identified by two or more unique peptides in only one experimental data set, 44 more proteins can be added to the list (Additional file 2: Tentatively identified egg white proteins), resulting in a total of 202 possibly identifications. Additional protein data, such as UniProt and RefSeq accession codes, number of identified peptides, sequence coverage, and protein PEP scores for accepted proteins (without contaminants) are provided in Additional file 3: Protein data. These results compared favorably with those obtained with peptide ligand library beads [11], where 68 proteins were identified with two or more sequence-unique peptides and a total of 148 proteins were obtained by accepting unique single peptide hits from different experiments (Figure 2). Furthermore, our study conservatively groups proteins with very similar sequences together and counts them as one "protein group", even when unique peptides pointed at the presence of isoforms or very similar proteins possibly encoded in different genes. Thus, the number of identified proteins is probably higher. A representative example is ovotransferrin, which seemed to represent a mixture of several forms containing many shared and a few unique peptides. Unique peptide data for accepted proteins (without contaminants), such as sequences, PEP scores, and distribution among gel sections are shown in Additional file 4: Peptide data.

Several previously identified proteins [9] were not identified immediately in the new egg white proteome. However, searching the new database version, IPIchick v3.65, with peptide sequences responsible for the previous identification of these proteins indicated that this was in many cases due to changes in the database. Thus, for instance, the ovosecretoglobulin sequence was no longer joined to a channel protein sequence in IPI00575434 but appeared with a new accession number, IPI00847051. Other proteins changed name. Thus, chondrogenesis-associated lipocalin (IPI00600353) is now lipocalin-type prostaglandin synthase D. The only proteins that could not be identified again in the present study were HMG-1 (IPI00595982), a hypothetical protein (IPI00597019), histone H1 (IPI00597019), 60S ribosomal protein L27 (IPI00577674) and poly(ADP-ribosyl) polymerase 1 (IPI00588387). The first two proteins were previously identified predominantly (HGM-1) or exclusively (Hypothetical protein) by in-solution tryptic cleavage, which was not performed in the present study. Three of these proteins, HMG-1, histone H1, and poly(ADP-ribosyl) polymerase were, however, confirmed in a recent study [11]. Therefore, the reason for their absence in the present study is not clear, but as these proteins are unlikely to play functional roles in egg white, their inclusion in egg white preparations may vary. Keratins were excluded from our results because they usually shared all or most peptides with common contaminants. Only few of the new egg white proteins identified using peptide ligand library beads [11] were also detected in the present study. These were nine proteins in the group of identifications with >2 unique peptides (Additional file 1: Egg white proteins identified with two or more unique peptides) and four among the tentatively identified proteins (Additional file 2: Tentatively identified egg white proteins).

**Figure 3:**
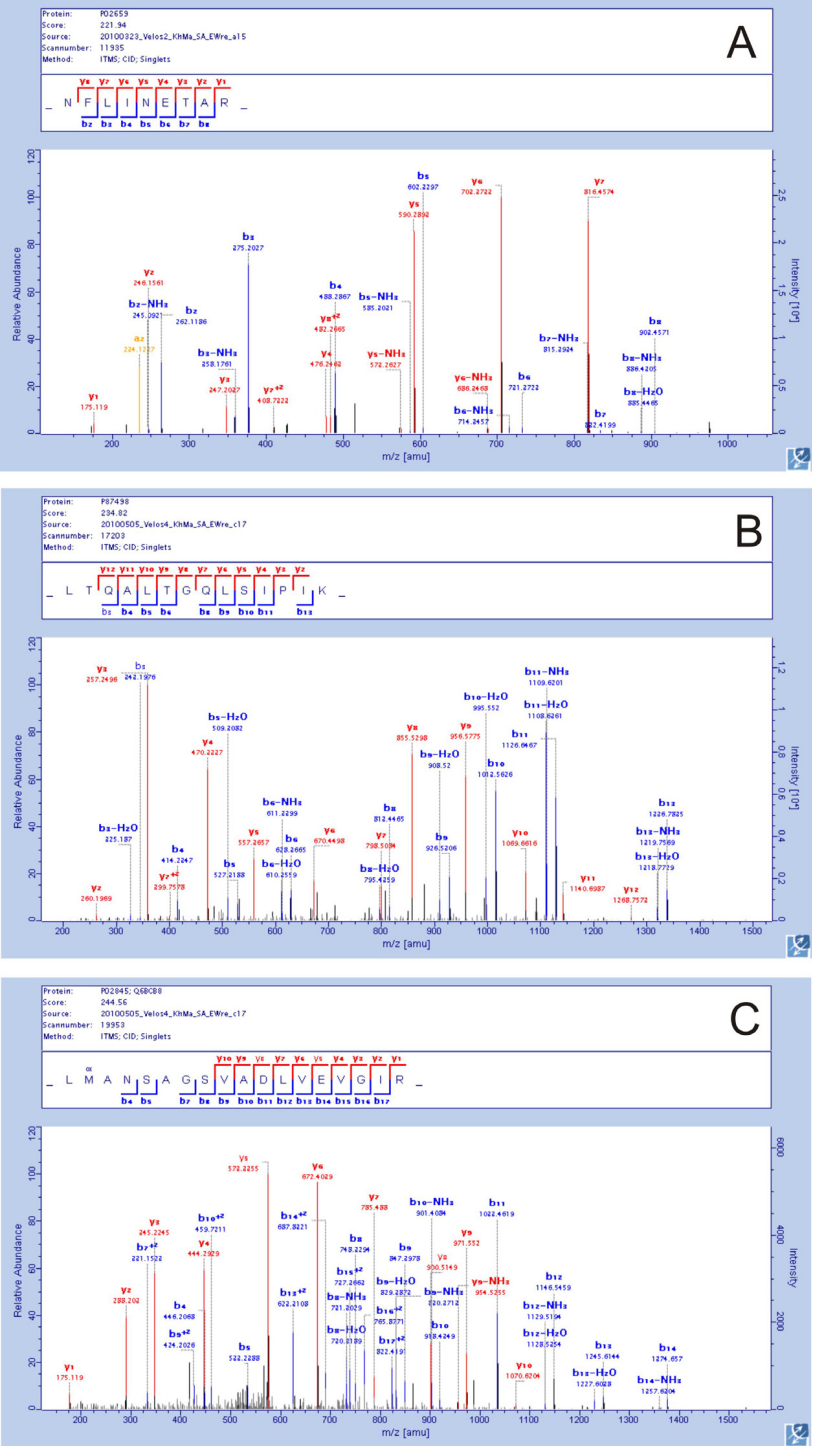
**Typical MaxQuant-annotated spectra instrumental in the identification of new egg white proteins**. **A**, apovitellenin-1 peptide corresponding to sequence positions 75-83. The precursor peptide mass of this doubly charged peptide was determined with a mass error of 0.35 ppm. The peptide posterior error probability (PEP) was 1.1E-15 and the MaxQuant score was 221. This is an example of a short peptide with almost uninterrupted b- and y-ion series. **B**, vitellogen-1 peptide corresponding to sequence positions 95-108. The precursor mass of this doubly charged peptide was determined with a mass error of 0.18 ppm. The PEP was 1.22E-28 and the MaxQuant score was 234. The most intense y-ion, y3, indicates the well known preferential cleavage N-terminal to proline. **C**, spectrum of a longer peptide corresponding to sequence positions 666-683 of vitellogenin-2. The precursor mass of this triply charged peptide was determined with a mass error of 0.53 ppm. PEP was 5.2E-42 and the score was 244. The fragmentation pattern shows long uninterrupted b- and y-ion series, but as frequently seen with CID fragmentation of longer peptides, the sequence coverage is not complete.

**Figure 4:**
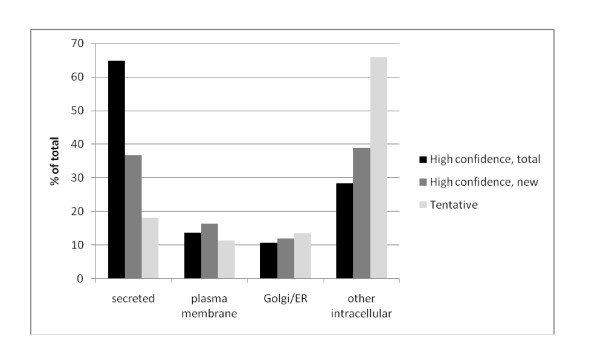
**Subcellular location of proteins identified in egg white**. Subcellular location of proteins as taken from the UniProt database (http://www.ebi.ac.uk/uniprot/) entry of the identified proteins, or of similar proteins identified by searching the database using FASTA (http://www.ebi.ac.uk/Tools/fasta33/), and from signal sequence predictions using SignalP (http://www.cbs.dtu.dk/services/SignalP/[24]) and InterProScan (http://www.ebi.ac.uk/Tools/InterProScan/). Proteins occurring in more than one cellular compartment are counted in each category.

Reassuringly, only two new protein identifications were contained among the 30 most abundant egg white proteins (Additional file 1: Egg white proteins identified with two or more unique peptides). This group of proteins contained 79 proteins that were not identified as egg white components previously. The new egg white proteins included several typical major yolk residents, such as apovitellinin-I, vitellogenin-1 to -3 and apolipoprotein B. These proteins are synthesized in the liver, carried to the ovary via the blood circulation, taken up by oocytes via receptor-mediated processes, and incorporated into the globular fraction of egg yolk [20]. Because the egg yolk was not damaged during mechanical separation of egg white and yolk, these proteins do not seem to be simple contaminants. Rather, residual protein not taken up by the egg cell may be liberated from the ovary together with the egg and migrate with the egg into the oviduct, mixing with egg white proteins secreted in the magnum section. In line with this suggestion, apovitellenin-I and vitellogenins have also been identified in the eggshell organic matrix [21]. This indicates that the oviduct fluid in the eggshell gland still contained these proteins. A few representative peptide fragmentation spectra for some of these proteins are shown in Figure 3. However, many of the new proteins present at low abundance are proteins normally found in intracellular compartments (Additional file 1: Egg white proteins identified with two or more unique peptides; Additional file 2: Tentatively identified egg white proteins). Golgi and ER proteins may have reached the oviduct fluid as by-products of the secretion of major egg white proteins. Other intracellular proteins may have come from damaged, leaky cells of the epithelium lining the oviduct, or from organelles, such as lysosomes, which occur in egg white [22]. Analysis of previously known subcellular locations of proteins identified in egg white shows a decrease in secreted proteins from approximately 64% in the whole proteome to 37% among the new proteins and 18% in tentative identifications (Figure 4). This is accompanied by a similar increase in intracellular proteins, indicating that we have now reached a depth of proteome characterization beyond which it may become difficult to identify functional egg white components. Therefore, minor specific egg white proteins of interest, such as MMP-2, may preferentially be enriched by specific methods before analysis [23]. However, the search for minor components in egg white remains of importance, because very low-abundance proteins, such as bone morphogenetic protein 1 (Additional file 1: Egg white proteins identified with two or more unique peptides) may have a biological role, for instance in early embryonic development.

## Conclusions

Our results indicate that current state of the art mass spectrometry technology is sufficiently advanced to permit direct mining of minor components of proteomes dominated by a few major proteins without the necessity to resort to broad specificity protein enrichment techniques, such as peptide ligand library tools, that change the proteome and render absolute quantification impossible. In addition we have significantly expanded the previously known egg white protein inventory.

## Abbreviations

CID: Collision-induced Decomposition; ESI: Electrospray Ionization; FDR: False Discovery Rate; FT: Fourier transform; IT: Ion Trap; LC: Liquid Chromatography; MALDI: Matrix-assisted Laser Desorption Ionization; MES: 2-(N-Morpholino) ethanesulfonic acid; PAGE: Polyacrylamide Gel Electrophoresis; PEP: Posterior Error probability; SDS: Sodium Dodecyl Sulphate; TOF: Time-of-flight.

## Competing interests

The authors declare that they have no competing interests.

## Authors' contributions

KM conceived the study, performed sample preparation and data acquisition. MM supplied methodological expertise. Both authors took part in the design of the study and were critically involved in manuscript drafting. All authors read and approved the final manuscript.

## Supplementary Material

Additional file 1 **Egg white proteins identified with two or more unique peptides**. Docx-file containing a list of proteins identified with high confidence. Click here for file

Additional file 2 **Tentatively identified egg white proteins**. Docx-file showing a list of proteins identified with one unique peptide in two of three experimental sets and proteins identified with 2 or more peptides in only one experimental set. Click here for file

Additional file 3 **Protein data**. Xls-file containing MaxQuant output data such as protein scores, number of peptides, Uniprot and Refseq accession codes, and sequence coverage. Click here for file

Additional file 4 **peptide data**. Xls-file containing MaxQuant output data such as peptide sequences, peptide scores, peptide mass and slice distribution. Click here for file

## References

[B1] Stevens L Egg white proteins Comp Biochem Physiol B 1991 100 1 9 10.1016/0305-0491(91)90076-P 1756612

[B2] Mine Y Recent advances in the understanding of egg white protein functionality Trends Food Sci Technol 1995 6 225 232 10.1016/S0924-2244(00)89083-4

[B3] Mine Y Kovacs-Nolan J Biologically active hen egg components in human health and disease J Poult Sci 2004 41 1 29 10.2141/jpsa.41.1

[B4] Mine Y Kovacs-Nolan J New insights in biologically active proteins and peptides derived from hen egg World Poult Sci 2006 62 87 95 10.1079/WPS200586

[B5] Anton M Nau F Nys Y Bioactive egg components and their potential uses World Poult Sci 2006 62 429 438 10.1017/S004393390600105X

[B6] Mine Y Egg proteins and peptides in human health - Chemistry, bioactivity and production Curr Pharmaceut Design 2007 13 875 884 10.2174/138161207780414278 17430187

[B7] Raikos V Hansen R Campbell L Euston SR Separation and identification of hen egg protein isoforms using SDS-PAGE and 2D gel electrophoresis with MALDI-TOF mass spectrometry Food Chem 2006 99 702 710 10.1016/j.foodchem.2005.08.047

[B8] Guérin-Dubiard C Pasco M Mollé D Désert C Croguennec T Nau F Proteomic analysis of hen egg white J Agric Food Chem 2006 54 3901 3910 16719513 10.1021/jf0529969

[B9] Mann K The chicken egg white proteome Proteomics 2007 7 3558 3568 10.1002/pmic.200700397 17722208

[B10] Boschetti E Righetti PG The art of observing rare protein species in proteomes with peptide ligand libraries Proteomics 2009 9 1492 1510 10.1002/pmic.200800389 19235170

[B11] D'Ambrosio C Arena S Scaloni A Guerrier L Boschetti E Mendieta ME Citterio A Righetti PG Exploring the chicken egg white proteome with combinatorial peptide ligand libraries J Proteome Res 2008 7 3461 3474 18570458 10.1021/pr800193y

[B12] Keidel EM Ribitsch D Lottspeich F Equalizer technology - Equal rights for disparate beads Proteomics 2010 10 2089 2098 10.1002/pmic.200900767 20340161

[B13] Olsen JV Schwartz JC Griep-Raming J Nielsen ML Damoc E Denisov E Lange O Remes P Taylor D Splendore M Wouters ER Senko M Makarov A Mann M Horning S A dual pressure linear ion trap-Orbitrap instrument with very high sequencing speed Mol Cell Proteomics 2009 8 2759 2769 10.1074/mcp.M900375-MCP200 19828875 PMC2816009

[B14] Cox J Hubner NC Mann M How much peptide sequence information is contained in ion trap tandem mass spectra? J Am Soc Mass Spectrom 2008 19 1813 1820 10.1016/j.jasms.2008.07.024 18757209

[B15] Cox J Mann M MaxQuant enables high peptide identification rates, individualized ppb-range mass accuracies and proteome-wide protein quantification Nature Biotechnol 2009 26 1367 1372 10.1038/nbt.1511 19029910

[B16] Cox J Mann M Computational Principles of determining and improving mass precision and accuracy for proteome measurements in an orbitrap J Am Soc Mass Spectrom 2009 20 1477 1485 10.1016/j.jasms.2009.05.007 19553133

[B17] Shevchenko A Tomas H Havlis J Olsen JV Mann M In-gel digestion for mass spectrometric characterization of proteins and proteomes Nature Protocols 2006 1 2856 2860 10.1038/nprot.2006.468 17406544

[B18] Rappsilber J Mann M Ishihama Y Protocol for micro-purification, enrichment, pre-fractionation and storage of peptides for proteomics using StageTips Nature Protocols 2007 2 1896 1906 10.1038/nprot.2007.261 17703201

[B19] Ishihama Y Oda Y Tabata T Sato T Nagasu T Rappsilber J Mann M Exponentially modified protein abundance index (emPAI) for estimation of absolute protein amount in proteomics by the number of sequenced peptides per protein Mol Cell Proteom 2005 4 1265 1272 10.1074/mcp.M500061-MCP200 15958392

[B20] Burley RW Vadehra DV The Avian Egg 1989 New York: John Wiley and Sons

[B21] Mann K Macek B Olsen JV Proteomic analysis of the acid-soluble organic matrix of the chicken calcified eggshell layer Proteomics 2006 6 3801 3810 10.1002/pmic.200600120 16767793

[B22] Yoon J Park J-M Kim K-J Kim Y-H Min J Antimicrobial activity of the cell organelles, lysosomes, isolated from egg white J Microbiol Biotechnol 2009 19 1364 1368 10.4014/jmb.0905.05053 19996688

[B23] Réhault-Godbert S Gautron J Labas V Belghazi M Nys Y Identification and characterization of the precursor of chicken matrix metalloproteases 2 (pro-MMP-2) in hen egg J Agric Food Chem 2008 56 6294 6303 18620399 10.1021/jf8003948

[B24] Emanuelsson O Brunak S von Heijne G Nielsen H Locating proteins in the cell using TargetP, SignalP, and related tools Nature Protocols 2007 2 953 971 10.1038/nprot.2007.131 17446895

