# Supplementary material for: In-depth analysis of the chicken egg white proteome using an LTQ Orbitrap Velos
Source: Proteome Sci. 2011 Feb 7;9:7. doi: 10.1186/1477-5956-9-7 (PMC3041730; doi:10.1186/1477-5956-9-7)
Supplement: Additional file 1 — Egg white proteins identified with two or more unique peptides. Docx-file containing a list of proteins identified with high confidence. [file 1477-5956-9-7-S1.DOCX]

**Egg white proteins identified with two and more unique peptides**

|  |  |  |  |  |  |  |
| --- | --- | --- | --- | --- | --- | --- |
| **No.** | **IPI Accession^1^** | **Protein** | **Experiment** | **Unique** ^3^  **peptides** | **Usual location** | **emPAI** |
|  |  |  |  |  |  |  |
| **1** | 00583974 **^2a,b^** | Ovalbumin; N-term acetylated | 1-3 | 37 | secreted | 251187.6 |
| **2** | 00683271 **^2a,b^**  00578012 **^2a,b^**  00821645 | Ovotransferrin  78kDa protein  105kDa protein  share 86 peptides with ovotransferrin | 1-3 | 98  5 | secreted | 37274.9 |
| **3** | 00600859 **^2a,b^** | Lysozyme C | 1-3 | 20 | secreted | 2309.1 |
| **4** | 00597129 **^2a,b^** | Ovomucoid | 1-3 | 19 | secreted | 773.3 |
| **5** | 00587313 **^2a,b^**  00681776 **^2a^** | Ovoinhibitor  53Kda protein; share 24 peptides | 1-3 | 25  1 | secreted | 561.3 |
| **6** | 00573738 **^2a,b^** | Ovalbumin-related protein Y; shares 1 peptide with IPI00585021 | 1-3 | 27 | secreted | 507.0 |
| **7** | 00585021 **^2a,b^** | Similar to ovalbumin-related protein Y (OVA-X); N-term. acetylated | 1-3 | 30 | secreted | 427.1 |
| **8** | 00600265 **^2a,b^** | Apolipoprotein D | 1-3 | 7 | secreted | 371.8 |
| **9** | 00847051 **^2a^** | Ovosecretoglobulin/10kDa protein | 1-3 | 3 | secreted | 315.2 |
| **10** | 00601265 **^2a,b^**  00822987 | Avidin  17kDa protein | 1-3 | 9 | secreted | 267.3 |
| **11** | 00589747 **^2a,b^**  00818180 | Ovostatin  164kDa protein; share 81 peptides | 1-3 | 82  4 | secreted | 250.2 |
| **12** | 00595847 **^2a,b^** | Similar to MGC82112 ; similar to α2-macroglobulin-like 1 | 1-3 | 82 | secreted | 219.4 |
| **13** | 00819356 **^2a,b^** | Clusterin/49kDa protein | 1-3 | 20 | secreted | 205.9 |
| **14** | 00584841 **^2a,b^** | Hep21 | 1-3 | 7 | secreted | 157.5 |
| **15** | 00576782 **^2a,b^** | Cystatin | 1-3 | 5 | secreted | 145.8 |
| **16** | 00600069 **^2a,b^**  00594673  00818310  00819914 | Extracellular fatty acid-binding protein  21kDa protein (80% identical to ExFABP)  Protein  20kDa protein | 1-3 | 8  4 | secreted | 137.9 |
| **17** | 00589360 **^2a,b^**  00822052 | Glutathione peroxidase 3  22kDa protein | 1-3 | 9 | secreted | 132.4 |
| **18** | 00600353 **^2a,b^** | Lipocalin-type prostaglandin D synthase/chondrogenesis –associated lipocalin | 1-3 | 7 | ER, Golgi, plasma membrane, secreted | 132.3 |
| **19** | 00584997 **^2a,b^** | Ovomucin α-subunit | 1-3 | 126 | secreted | 101.6 |
| **20** | 00598113 **^2a,b^** | Ovoglycoprotein | 1-3 | 8 | secreted | 99.0 |
| **21** | 00580509 | Apovitellenin-1 | 1-3 | 5 | secreted | 99.0 |
| **22** | 00578016 **^2a,b^** | Dickkopf-related protein 3 | 1-3 | 18 | secreted | 65.6 |
| **23** | 00574804 **^2a,b^** | Gallinacin-11 (VMO-II; β-defensin-11) | 1-3 | 6 | secreted | 62.1 |
| **24** | 00598229 **^2a,b^**  00819823 | Tenp  44kDa protein; share 11 of 15 peptides | 1-3 | 15  4 | secreted | 60.1 |
| **25** | 00574195 **^2a,b^** | Serum albumin (α-livetin) | 1-3 | 30 | secreted | 46.5 |
| **26** | 00597482 **^2a,b^** | (Similar to) ovomucin β-subunit | 1-3 | 53 | secreted | 30.6 |
| **27** | 00596061 **^2a,b^** | VMO-I | 1-3 | 10 | secreted | 27.5 |
| **28** | 00681274 **^2a,b^**  00822809 | Gallin  Similar to meleagrin | 1-3 | 3 | secreted | 20.5 |
| **29** | 00573473 **^2a,b^** | Hypothetical protein; partial hemopexin | 1-3 | 8 | secreted | 16.8 |
| **30** | 00847036 | 8kDa protein/secretory trypsin inhibitor | 1-3 | 2 | secreted | 9.0 |
| **31** | 00577639 **^2a^** | Hypothetical protein; partial serpin domain; some similarity to part of plasma protease C1 inhibitor | 1-3 | 2 | secreted | 9.0 |
| **32** | 00589837 **^2a^** | Similar to autotoxin isoform 1/ ectonucleotide pyrophasphatase/ phosphodiesterase family member 1 | 1-3 | 25 | secreted/plasma membrane | 9.0 |
| **33** | 00590040 **^2a,b^** | Lymphocyte antigen 86 | 1-3 | 5 | secreted | 9.0 |
| **34** | 00593624 **^2b^** | Similar to Kazal-type serine protease inhibitor | 1-3 | 2 | secreted | 9.0 |
| **35** | 00655503 **^2a,b^**  00837485  00582169 | Actin, cytoplasmic 1; shares most of its peptides with other chicken actin entries and bovine actin, a common contaminant, which is 100% identical.  Actin, cytoplasmic 2;  Actin, alpha skeletal muscle  Share 14 of 15 and 6 of 7 peptides with IPI00655503 | 1-3 | 15  1  1 | cytoplasm | 9.0 |
| **36** | 00591488 **^2a,b^** | Ig µ chain C region | 1-3 | 8 | secreted | 7.7 |
| **37** | 00810881 **^2a,b^** | Similar to complement component 8, γ polypeptide | 1-3 | 8 | secreted | 7.4 |
| **38** | 00594746 **^2a,b^** | Riboflavin-binding protein | 1-3 | 8 | secreted | 7.1 |
| **39** | 00571581 **^2a,b^** | Sulfhydryl oxidase 1 | 1-3 | 19 | Golgi membrane | 7.1 |
| **40** | 00576977 **^2a^**  00819077  00572919  00598581 | 12kDa protein  12kDa protein  Histone H4  Histone H4 typeVIII | 1-3 | 5 | nucleus | 6.2 |
| **41** | 00587452 **^2a^** | Similar to oncoprotein-induced transcript 1 | 1-3 | 8 | secreted | 5.8 |
| **42** | 00602226  00591299 | Vitellogenin-2  205kDa protein; share 49 of 50 peptides | 1-3 | 50  1 | secreted | 5.4 |
| **43** | 00586548 **^2a,b^**  00601120 | Angiopoietin-like protein 3  54kDa protein,  97.6% identical, share 11 peptides | 1-3 | 13  1 | secreted | 5.1 |
| **44** | 00581002 **^2a,b^**  00821431 | Ubiquitin  52kDa protein | 1-3 | 2 | cytoplasm | 4.6 |
| **45** | 00578622 **^2b^**  00821633 | Ovocalyxin-32 | 1-3 | 2 | secreted | 4.2 |
| **46** | 00866802 **^2a,b^** | Polymeric immunoglobulin receptor | 1-3 | 13 | plasma membrane, secreted | 4.1 |
| **47** | 00818415  00583337 **^2a,b^** | 24kDa protein  Peptidyl-prolyl-cis/trans isomerase B | 1-3 | 5 | ER lumen | 4.0 |
| **48** | 00600127 **^2b^** | Similar to bactericidal permeability-increasing protein | 1-3 | 10 | secreted | 4.0 |
| **49** | 00597109 **^2a,b^** | Aminopeptidase Ey | 1-3 | 19 | plasma membrane, secreted | 3.9 |
| **50** | 00597264 **^2b^**  00573025 | Hypothetical protein; Pleiotrophin  15kDa protein; Midkine | 1-3 | 3 | secreted | 3.6 |
| **51** | 00593541 **^2b^** | Renin/prorenin receptor | 1-3 | 7 | plasma membrane | 3.6 |
| **52** | 00597105 **^2a^** | Similar to METRNL (meteorin-like) protein | 1-3 | 7 | secreted | 3.3 |
| **53** | 00580429 | 13kDa protein; similar to junction plakoglobin; shares 1 of 5 peptides with IPI00579090 (β-catenin) | 1-3 | 4 | cytoplasm, plasma membrane | 3.2 |
| **54** | 00595270 **^2a,b^** | TIMP-2 | 1-3 | 5 | secreted | 3.2 |
| **55** | 00583507 | Putative uncharacterized protein; similar to syntenin-1 | 1-3 | 4 | ER membrane, plasma membrane | 3.2 |
| **56** | 00822431 | Histone H2B variants | 1-3 | 3 | nucleus | 3.2 |
| **57** | 00574703 **^2a^**  00683203 **^2a^** | 10kDa protein; Ig λ chain  10kDa protein; shares 1 of 2 peptides | 1-3 | 3  1 | secreted | 3.0 |
| **58** | 00598054 **^2a^** | Immunoglobulin J polypeptide | 1-3 | 3 | secreted | 3.0 |
| **59** | 00595834 **^2a^** | FAMC3 protein | 1-3 | 4 | secreted | 2.8 |
| **60** | 00600609  00684262  00812261 **^2a^**  00594704  00819112  00819112 | 13kDa protein; Ig superfamily  13kDa protein  Similar to Ig α chain  7kDa protein  15kda protein  15kDa protein | 1-3 | 2  1  1  1  1  1 | secreted | 2.7 |
| **61** | 00580810 **^2a,b^** | Similar to tazarotene-induced gene 2 | 1,3 | 5 | secreted | 2.6 |
| **62** | 00575584 | α-Enolase; shares 2 peptides with IPI00592520 (γ-Enolase) | 1-3 | 5 | cytoplasm | 2.4 |
| **63** | 00576813 | Putative uncharacterized protein/similar to palmitoyl protein thioesterase 1 | 1,3 | 5 | lysosome | 2.2 |
| **64** | 00599307 | 60S acidic ribosomal protein P1; N-term acetylated | 1,3 | 3 | cytoplasm | 2.2 |
| **65** | 00587380 **^2a,b^**  00582445 | Cytotactin/tenascin; share 27 0f 28 peptides | 1-3 | 28 | secreted | 2.1 |
| **66** | 00589382 **^2a,b^** | Hypothetical protein; similar to acyloxyacyl hydrolase | 1-3 | 10 | secreted | 2.0 |
| **67** | 00680973 **^2b^**  00583269 **^2b^**  00599848  00602067 | CEPU-1, isoform1  CEPU-1  CEPU-1, isoform 2  Neural secreted glycoprotein | 1-3 | 4 | secreted | 1.9 |
| **68** | 00822414 | 15kDa selenoprotein | 1-3 | 2 | ER lumen | 1.7 |
| **69** | 00589985 | Elongation factor 1-α 1 | 1-3 | 5 | cytoplasm | 1.7 |
| **70** | 00581589 **^2a,b^**  00593580 **^2a,b^** | 135kDa protein; similar to PIT 54  similar to deleted in malignant brain tumors 1; share 5 peptides | 1-3 | 12  1 | secreted/membrane | 1.5 |
| **71** | 00820456 **^2a^**  00595705 | 16kDa protein/Epididymal secretory protein E1 (Niemann-Pick disease type C2 protein) | 1-3 | 3 | secreted | 1.5 |
| **72** | 00591843  00820086 | Vitellogenin-1  211kDa protein; share 30 of 31 peptides | 1-3 | 31  1 | secreted | 1.5 |
| **73** | 00578632  00820116 | 14-3-3 Protein ζ (zeta)  24kDa protein  Share 1 of 5 peptides with other 14-3-3 proteins | 1-3 | 4 | cytoplasm | 1.4 |
| **74** | 00596201 **^2a,b^** | Olfactomedin-like protein 3 | 1-3 | 7 | secreted | 1.4 |
| **75** | 00588294 **^2a,b^** | Similar to transmembrane protease, serine 9 | 1-3 | 16 | plasma membrane | 1.4 |
| **76** | 00574658 **^2a,b^** | TIMP-3 | 1-3 | 4 | secreted | 1.3 |
| **77** | 00599180 | Hypothetical protein; leucine-rich repeat-containing protein | 1-3 | 3 | plasma membrane | 1.3 |
| **78** | 00580166 **^2a,b^** | Glutathione S-transferase 2 | 1-3 | 5 | cytoplasm | 1.3 |
| **79** | 00593199 | β-2-Microglobulin | 1,3 | 2 | secreted | 1.2 |
| **80** | 00577274 | RGD-CAP; similar to transforming growth factor-induced protein iq-h3 | 1-3 | 8 | secreted | 1.2 |
| **81** | 00587277 | Similar to SDF3 (PEDF-like, serpin-family) | 1-3 | 8 | secreted | 1.2 |
| **82** | 00775749  00819590 | Apolipoprotein B  51kDa protein; shares 5 peptides with Apo B | 1-3 | 75  1 | secreted | 1.1 |
| **83** | 00595826 **^2a,b^**  00822310  00819712 | Golgi apparatus protein 1  130kDa protein  120kDa protein | 1-3 | 15 | ER membrane | 1.1 |
| **84** | 00820677  00578917 | 60kDa protein  Cochlin | 1-3 | 7 | secreted | 1.0 |
| **85** | 00600823 | Proteasome subunit α type-7 | 1-3 | 3 | cytoplasm | 1.0 |
| **86** | 00580765 **^2a,b^** | Apolipoprotein A-I | 1-3 | 5 | secreted | 0.9 |
| **87** | 00585627 **^2a,b^** | Similar to bactericidal/permeability-increasing protein like-2 | 1,3 | 4 | secreted | 0.9 |
| **88** | 00588908 **^2a^** | Similar to ovulatory protein 2 | 1-3 | 3 | secreted | 0.9 |
| **89** | 00588868 | Eukaryotic initiation factor 4A-II | 1,3 | 4 | cytoplasm | 0.9 |
| **90** | 00681344 **^2b^** | Similar to Kunitz-like protease inhibitor | 1-3 | 3 | secreted | 0.9 |
| **91** | 00590375 **^2b^** | Glucose-regulated 78kDa protein; HSP 70 family, shares 2 of 8 peptides with IPI00582091 | 1-3 | 6 | ER membrane (lumen) | 0.9 |
| **92** | 00829402 | ASPIC/cartilage acidic protein 1 | 1-3 | 9 | secreted | 0.9 |
| **93** | 00582452 | Triosephosphate isomerase | 1-3 | 3 | cytoplasm | 0.9 |
| **94** | 00597853 | Proteasome subunit, α-type | 2,3 | 3 | cytoplasm | 0.8 |
| **95** | 00602683 | 20kDa protein; similar to putative Agr-rich protein mutated in early tumors | 1,3 | 2 | secreted | 0.8 |
| **96** | 00584676 | Similar to corticotrophin-releasing factor-binding protein | 1,3 | 3 | secreted | 0.8 |
| **97** | 00685019 **^2a,b^**  00577021 | 24kDa protein  Ig λ chain C-region | 1-3 | 3 | secreted | 0.8 |
| **98** | 00820598  00810914  00571613 | 26kDa protein; FAMB3 family  Hypothetical protein; FAMB3 family  26kDa protein; FAM3B family | 1,2 | 2 | secreted | 0.8 |
| **99** | 00813945  00883281  00814509 **^2b^** | Similar to SLIT2 isoform 1  169kDa protein  Similar to SLIT2 isoform 2 | 1-3 | 12 | secreted | 0.7 |
| **100** | 00574055 **^2a,b^** | Procollagen-lysine, 2-oxoglutarate 5-dioxgenase 1 | 1-3 | 7 | ER lumen | 0.7 |
| **101** | 00572503 | Similar to α-mannosidase class 2B, member 2 | 1-3 | 7 | secreted | 0.7 |
| **102** | 00822785 **^2a,b^** | 22kDa protein/ADP-ribosylation factor 5 | 1,3 | 4 | Golgi, cytoplasm | 0.7 |
| **103** | 00581527  00823322 | Calmodulin; N-term acetylated | 1-3 | 2 | cytoplasm | 0.7 |
| **104** | 00596673 | Protein disulfide-isomerase | 1-3 | 6 | ER membrane, plasma membrane | 0.7 |
| **105** | 00598551 | Similar to prosomal P27K protein/proteasome subunit α-type | 1-3 | 2 | cytoplasm | 0.6 |
| **106** | 00572470 | Annexin I | 1-3 | 2 | nucleus, cytoplasm, plasma membrane | 0.6 |
| **107** | 00593654 | Hypothetical protein; similar to di-N-acetylchitobiase | 1-3 | 3 | lysosome | 0.6 |
| **108** | 00585747 | Elongation factor 2 | 1,3 | 8 | cytoplasm | 0.6 |
| **109** | 00580059  00812933 | Peroxiredoxin-1  Similar to natural killer cell enhancing factor | 1-3 | 2 | cytoplasm | 0.6 |
| **110** | 00582091 | HSP70; shares 2 of 6 peptides with IPI00590375 (78kDa glucose-regulated protein) | 1,3 | 4 | cytoplasm, cell surface | 0.6 |
| **111** | 00598223 **^2b^** | Similar to decoy receptor 3 | 2,3 | 2 | secreted | 0.5 |
| **112** | 00575271 | Protein disulfide isomerase A3 | 1-3 | 5 | ER lumen | 0.5 |
| **113** | 00822110  00579245 | 105kDa protein; similar to insulin-degrading enzyme  102kDa protein; similar to insulin-degrading enzyme | 2,3 | 5 | cytoplasm, plasma membrane | 0.5 |
| **114** | 00580985 | Calreticulin | 1-3 | 3 | ER lumen | 0.5 |
| **115** | 00590239 | 40S ribosomal protein SA/laminin receptor 1 | 2,3 | 2 | plasma membrane, cytoplasm, nucleus | 0.5 |
| **116** | 00572461 | α-Actinin-4 | 2,3 | 8 | cytoskeleton | 0.5 |
| **117** | 00590085 | Hypothetical protein; similar to galactocerebrosidase | 1,3 | 2 | lysosome | 0.5 |
| **118** | 00593765 | Putative uncharacterized protein; similar to gastric intrinsic factor/transcobalamin | 1-3 | 2 | secreted | 0.5 |
| **119** | 00603219  00819358 | Tumor necrosis factor-related apoptosis-inducing ligand | 1,3 | 2 | plasma membrane | 0.4 |
| **120** | 00573327 | Vitamin D-binding protein | 1,3 | 2 | secreted | 0.4 |
| **121** | 00577371 | Hypothetical protein; thiol protease family | 1-3 | 3 | secreted, lysosome | 0.4 |
| **122** | 00599064 | Putative uncharacterized protein; similar to N-acylsphingosine amidohydrolase | 1,3 | 3 | lysosome | 0.4 |
| **123** | 00574951 | Semaphorin-3C | 2,3 | 5 | secreted | 0.4 |
| **124** | 00570770 | Endoplasmin; shares 1 of 6 peptides with IPI00820593 | 1-3 | 5 | ER lumen | 0.4 |
| **125** | 00602986 **^2a^** | Similar to desmoplakin isoform II isoform 2 | 1-3 | 15 | cell junction | 0.4 |
| **126** | 00818934  00823141 | 184kda protein; vitellogenin-3 | 1,3 | 12 | secreted | 0.4 |
| **127** | 00575767 | Catalase | 1-3 | 4 | peroxysome | 0.4 |
| **128** | 00602351 | Hepatic α-amylase | 1-3 | 4 | secreted | 0.4 |
| **129** | 00586802 | Hypothetical protein; transitional ER ATPase | 1-3 | 5 | cytoplasm | 0.4 |
| **130** | 00593455  00575514 | Cadherin-1  Hypothetical protein; similar to cadherin | 1-3 | 3 | plasma membrane | 0.3 |
| **131** | 00601768 **^2a,b^** | Angiotensin-converting enzyme | 1,3 | 6 | secreted | 0.3 |
| **132** | 00582281 | Similar to β-hexosaminidase β-chain | 1,3 | 5 | lysosome | 0.3 |
| **133** | 00584670 | Similar to prostatic acid phosphatase | 1,3 | 2 | secreted, plasma membrane, lysosome | 0.3 |
| **134** | 00592552 | Bone morphogenetic protein 1 | 1-3 | 5 | secreted | 0.3 |
| **135** | 00576308 **^2a^** | Similar to carboxypeptidase D | 1-3 | 7 | plasma membrane | 0.3 |
| **136** | 00597023 | Hypothetical protein; similar to legumain | 2,3 | 2 | lysosome | 0.3 |
| **137** | 00596315 | Guanine nucleotide-binding protein subunit β2-like 1 | 2,3 | 2 | cytoplasm | 0.3 |
| **138** | 00570697  00818683 | Histidine ammonia lyase  71kDa protein | 1-3 | 3 | cytoplasm | 0.3 |
| **139** | 00582635 **^2a^** | Similar to aminopeptidase A | 1-3 | 5 | plasma membrane | 0.3 |
| **140** | 00819050 | Similar to ceruloplasmin | 1-3 | 3 | secreted | 0.2 |
| **141** | 00577039 | Annexin A2 | 1-3 | 2 | cytoplasm, plasma membrane, secreted | 0.2 |
| **142** | 00682229 | 66kDa protein; similar to UDP-N-acetyl-α-D-galactosamine:polypeptide N-acetylgalactosaminyltransferase 4 | 1,3 | 2 | Golgi membrane | 0.2 |
| **143** | 00579090  00822777 | β-catenin  75kDa protein  share 1 of 4 peptides with IPI00580429 | 1-3 | 3 | plasma membrane | 0.2 |
| **144** | 00583980 | 195kDa protein; C3 and PZP-like, alpha-2-macroglobulin domain containing protein 8  8 | 1-3 | 5 | secreted | 0.2 |
| **145** | 00818621  00591231 | 57kDa protein; similar to ER-Golgi intermediate compartment 53kDa protein | 1,3 | 2 | ER/Golgi membrane | 0.2 |
| **146** | 00589584 | RAB-GDP dissociation inhibitor | 1,3 | 3 | cytoplasm | 0.2 |
| **147** | 00587219 | Lamin-A | 1-3 | 2 | cytoplasm/cytoskeleton | 0.2 |
| **148** | 00581368 | Ovocleidin-116 | 1,2 | 2 | secreted | 0.2 |
| **149** | 00819496  00573261 | 98kDa protein; similar to cell death 6-interacting protein  Putative uncharacterized protein; domain:BRO1 | 1,2 | 4 | intracellular | 0.2 |
| **150** | 00821924 | Aminopeptidase, puromycin sensitive | 1,3 | 2 | cytoplasm, nucleus | 0.1 |
| **151** | 00681096 | Similar to complement C4-1 | 2,3 | 2 | secreted | 0.1 |
| **152** | 00596586 | HSP90-α; shares 1 of 3 peptides with IPI00820593 (HSP90-β) | 1-3 | 2 | cytoplasm | 0.1 |
| **153** | 00570607  00819130 | Hypoxia up-regulated protein 1  111kDa protein | 1-3 | 2 | ER lumen | 0.1 |
| **154** | 00591552  00820593 | Heat shock cognate protein HSP90 β  84kDa protein  Share 1 peptide of 5 with IPI00570770 (endoplasmin), and 1 peptide of 5 with IPI00596673 (HSP90 α) | 1-3 | 3 | cytoplasm | 0.1 |
| **155** | 00585604 | 189kDa protein; similar to mucin 5 | 2,3 | 3 | secreted | 0.1 |
| **156** | 00583368  00818136 | Similar to procollagen-lysine, 2-oxoglutarate 5-dioxygenase; 83kDa protein | 1,3 | 2 | ER membrane (lumen) | 0.1 |
| **157** | 00575808  00822939 | Putative uncharacterized protein; ATP-citrate synthase  121kDa protein; | 1,3 | 2 | cytoplasm | 0.1 |
| **158** | 00572165 | Myosin-9 | 1,3 | 4 | cytoskeleton | 0.1 |
|  |  |  |  |  |  |  |

Entries are ordered according to decreasing emPAI. **^1^**, only the IPI accession number of the leading protein (the protein with most identified peptides) of a group of similar proteins is given. Complete data are shown in Supplementary file 3. **^2a,b^**, previously identified in [9]**^a^** or [11]**^b^**. **^3^**, protein group unique peptides.
