# Supplementary material for: In-depth analysis of the chicken egg white proteome using an LTQ Orbitrap Velos
Source: Proteome Sci. 2011 Feb 7;9:7. doi: 10.1186/1477-5956-9-7 (PMC3041730; doi:10.1186/1477-5956-9-7)
Supplement: Additional file 2 — Tentatively identified egg white proteins. Docx-file showing a list of proteins identified with one unique peptide in two of three experimental sets and proteins identified with 2 or more peptides in only one experimental set. [file 1477-5956-9-7-S2.DOCX]

**Tentatively identified egg white proteins**

|  |  |  |  |  |  |  |
| --- | --- | --- | --- | --- | --- | --- |
| **No.** | **IPI Accession^1^** | **Protein** | **Experiment** | **Unique**  **Peptides ^3^** | **Location** | **emPAI** |
|  |  |  |  |  |  |  |
| **1** | 00683104 **^2b^**  00590686 **^2a^** | Histone H2A  Histone H2A-V  share 1 peptide of 2 with many HA2 variants | 1-3 | 1  1 | nucleus | 2.2 |
| **2** | 00883091 | Plakoglobin fragment | 1-3 | 1 | cell junction | 2.2 |
| **3** | 00576073 | Hypothetical protein; similar to THO complex subunit 4 | 1 | 2 | nucleus, cytoplasm | 1.6 |
| **4** | 00574447  00651224  00575899  00684128  00683452 | Histone H3.3  Histone H3  16kDa protein  16kDa protein  Histone H3.2; entries share 1 of 2 peptides | 1,3 | 1  1 | nucleus | 1.2 |
| **5** | 00587398 | Actin-related protein 3 | 3 | 4 | cytoskeleton | 1.0 |
| **6** | 00591852  00577739  00579092  00820692  00818270 | 14-3-3 protein beta/alpha  14-3-3 protein theta/gamma  14-3-3 Protein epsilon  29kDa protein  26kDa protein | 1-3 | 2  1  1  1  1 | cytoplasm | 0.9 |
| **7** | 00812585 ^2a^ | Similar to immunoglobulin λ chain | 1,2 | 1 | secreted | 0.8 |
| **8** | 00603587  00822443 | Hypothetical protein/α-tubulin 8  44kDa protein/α-tubulin 2  share 3-4 peptides with each other and bovine α-tubulin 1, a common contaminant | 1,3 | 1  1 | cytoskeleton | 0.7 |
| **9** | 00580626  00592950  00603718 | β-Tubulin 3/7  β-Tubulin 1/2  β-tubulin 4; share most peptides | 1,3 | 1  1  1 | cytoskeleton | 0.7 |
| **10** | 00590301 | Voltage-dependent anion channel 1 | 3 | 4 | plasma membrane | 0.7 |
| **11** | 00600819 | Collagen α-1 (X) chain | 1,3 | 1 | secreted | 0.6 |
| **12** | 00592949 | Proteasome subunit, α-type | 3 | 2 | cytoplasm | 0.6 |
| **13** | 00593872 | Proteasome α-2 subunit | 3 | 2 | cytoplasm | 0.6 |
| **14** | 00581952 | Putative uncharacterized protein/tropomyosin | 3 | 2 | cytoskeleton | 0.4 |
| **15** | 00572756 **^2b^** | Ovocleidin-17 | 1-3 | 1 | secreted | 0.4 |
| **16** | 00577421 | HSP-60, mitochondrial | 1 | 2 | mitochondrion | 0.4 |
| **17** | 00580968 | T-complex protein 1, subunit eta | 1 | 3 | cytoplasm | 0.4 |
| **18** | 00651336 | Putative uncharacterized protein/heterogeneous nuclear ribonucleoprotein A2/B1 | 1 | 2 | nucleus | 0.3 |
| **19** | 00591422  00571469 | F-actin-capping protein subunit beta isoforms 1 and 2 | 3 | 2 | cytoskeleton | 0.3 |
| **20** | 00576279 | Putative uncharacterized protein; similar to eIF6 | 2,3 | 1 | cytoplasm, nucleus | 0.3 |
| **21** | 00586516 **^2b^**  00684903 | Similar to human P5 isoform 2 (protein disulfide isomerase)  Putative uncharacterized protein (fragment) | 2,3 | 1 | secreted/membrane | 0.3 |
| **22** | 00589224 | Similar to maspin (serpin protease inhibitor family) | 1-3 | 1 | cytoplasm, secreted | 0.3 |
| **23** | 00601033 | L-Lactate dehydrogenase B chain | 1,3 | 2 | cytoplasm | 0.3 |
| **24** | 00576601 | Putative uncharacterized protein; similar to heterogeneous nuclear riboprotein | 1,3 | 2 | nucleus | 0.3 |
| **25** | 00594653 | Glyceraldehyde-3-phosphate dehydrogenase; shares 1 of 2 peptides with the bovine enzyme (common contamination) | 1-3 | 1 | cytoplasm | 0.3 |
| **26** | 00573727 **^2b^**  00820954 | Putative uncharacterized protein; similar to nucleobindin  49kDa protein; similar to nucleobindin | 3 | 2 | cytoplasm, plasma membrane, secreted | 0.2 |
| **27** | 00573643  00571478  00820847 | Hypothetical protein  Ras-related protein Rab-6a  21kDa protein | 1-3 | 1 | Golgi apparatus | 0.2 |
| **28** | 00682113  00574064 | 58kDa protein  Pyruvat kinase muscle isozyme | 3 | 3 | cytoplasm | 0.2 |
| **29** | 00581163 | Similar to carboxypeptidase E | 1 | 2 | secreted | 0.2 |
| **30** | 00597100 | Hypothetical protein; similar to transcriptional adapter 2B | 1-3 | 1 | nucleus | 0.2 |
| **31** | 00592330 | Putative uncharacterized protein/HSP70 protein 13 | 1,3 | 2 | ER | 0.2 |
| **32** | 00584371 | Proactivator polypeptide | 1,2 | 2 | lysosome | 0.2 |
| **33** | 00575877  00822490 | 122kDa protein  Contactin-1 | 3 | 2 | plasma membrane | 0.1 |
| **34** | 00581158 | Complement C3 | 1 | 2 | secreted | 0.1 |
| **35** | 00583347 | Similar to N-acetylglucosaminyl transferase V | 1,3 | 1 | Golgi membrane | 0.1 |
| **36** | 00592520 | γ-Enolase; shares 2 of 3 peptides with α-enolase | 1-3 | 1 | cytoplasm | 0.1 |
| **37** | 00593450 | Hypothetical protein/UDP-glucose ceramide glucosyltransferase-like 1 | 1 | 2 | ER lumen | 0.1 |
| **38** | 00594476 | DNAJ homolog subfamily C, member 3 | 1,2 | 1 | ER | 0.1 |
| **39** | 00594512  00822376 | Similar to UTP14, U3 small nucleolar ribonucleoprotein, homolog A/15kDa protein | 1,2 | 1 | nucleus | 0.1 |
| **40** | 00597128 | Similar to UDP-N-acetyl-α-D-galactosamine: Polypeptide N-acetylgalactosaminyltransferase 12 | 1-3 | 1 | Golgi membrane | 0.1 |
| **41** | 00604251 | Similar to tRNA splicing endonuclease 54 homolog | 1-3 | 1 | nucleus | 0.1 |
| **42** | 00573263  00820606 | Similar to bullous pemphogoid antigen 1  620kDa protein | 2,3 | 1 | cytoskeleton | <0.1 |
| **43** | 00786274 | PAR3 | 1,2 | 1 | cell junctions | <0.1 |
| **44** | 00591842 | Similar to fat tumor suppressor 1 | 1 | 2 | plasma membrane | <0.1 |
|  |  |  |  |  |  |  |

This table contains egg white proteins identified with one unique peptide in several experiments, with several unique peptides in one experimental set, or with only one of two unique peptides validated manually. Entries are ordered according to decreasing emPAI. **^1^**, only the IPI accession number of the leading protein (the protein with most identified peptides) of a group of similar proteins is given. Complete data are shown in Supplementary file 3. **^2a,b^**, previously identified in [9]**^a^** or [11]**^b^**. **^3^**, group unique peptides.
